# Glutamate receptors function as scaffolds for the regulation of β-amyloid and cellular prion protein signaling complexes

**DOI:** 10.1186/s13041-015-0107-0

**Published:** 2015-03-24

**Authors:** Alison Hamilton, Gerald W Zamponi, Stephen S G Ferguson

**Affiliations:** J. Allyn Taylor Centre for Cell Biology, Robarts Research Institute, University of Western Ontario, 100 Perth Dr, London, Ontario N6A 5 K8 Canada; Department of Physiology & Pharmacology, University of Western Ontario, London, Ontario Canada; Department of Physiology & Pharmacology, Hotchkiss Brain Institute, Cumming School of Medicine, University of Calgary, Calgary, Alberta Canada

**Keywords:** Alzheimer’s disease, β-amyloid, Cellular prion protein, Glutamate, APPswe/PS1ΔE9, Metabotropic glutamate receptor, NMDA, Neurodegeneration

## Abstract

Alzheimer’s disease (AD) is a progressive neurodegenerative disorder that affects 36 million people worldwide, but currently has no effective treatment options. One of the original hallmarks of AD are plaques comprised of beta amyloid (Aβ) and neurofibrillary tangles comprised of phosphorylated Tau protein. However, it is soluble oligomeric Aβ which is more closely correlated with cognitive decline and is therefore considered to be the neurotoxic species. Oligomeric Aβ has recently been shown to form complexes with the glycosylphosphatidylinositol (GPI)-anchored membrane protein, cellular prion protein (PrP^c^), and these complexes are believed to play an important role in the progression of AD pathogenesis. Glutamate, the major excitatory neurotransmitter is responsible for mediating learning and memory under normal physiological conditions. However, the dysregulation of glutamatergic signaling has also been implicated in a number of neurodegenerative diseases including AD. Glutamate acts via both ionotropic glutamate receptors (iGluR) and metabotropic glutamate receptors (mGluR), each of which have been implicated in AD. There is now growing evidence to suggest that mGluR5 may contribute the AD pathogenesis by acting as scaffolds for the PrP^c^/Aβ oligomer complex, enabling the propagation of neurotoxic signaling in AD. In addition, PrP^c^ and Aβ oligomer signaling via NMDARs may also contribute to AD pathology. The current review overviews our current understanding of the role of PrP^c^ and Aβ oligomers in regulating glutamate receptor signaling, as well as highlights the importance of understanding these signaling complexes to develop more effective therapeutic strategies to treat AD.

Alzheimer’s disease (AD) is a progressive neurodegenerative disorder that is the leading cause of dementia affecting people over 50 years of age. There are approximately 36 million people worldwide with AD, and this number is expected to double by 2050, as the consequence of an aging world population [1]. Patients with AD suffer from memory loss and cognitive decline, which increases in severity as the disease progresses. Despite the rapidly growing incidence of AD there is no cure, and current therapeutic strategies have limited efficacy [1]. Pathophysiologically, the AD brain undergoes severe shrinkage that is caused by extensive synaptic and neuronal loss [2]. The original pathological features of AD were characterised by Alois Alzheimer in post mortem examination of patients suffering from severe memory loss [3,4]. These hallmarks include fibrillar plaques primarily composed of the protein beta amyloid (Aβ) and neurofibrillary tangles (NFTs) of hyperphosphorylated tau protein [2,5]. However, additional AD markers include inflammation, characterized by activated microglia, which have been shown to cluster in brain regions showing substantial neurodegeneration [6] and oxidative stress, which results from an imbalance of reactive oxygen species (ROS) and antioxidants [7,8]. Recent studies have shown that the metabotropic glutamate receptor 5 (mGluR5) antagonist MTEP and mGluR5 knockout is protective in AD mice and that the N-methyl-D-aspartate (NMDA) receptor antagonist may be protective at low concentrations [9,10]. The present review will overview the contribution of β-amyloid and cellular prion protein (PrP^C^) in the regulation of mGluR5 and NMDA receptor signaling.

## β-amyloid and PrP^C^

β-amyloid is a 39–43 amino acid peptide that is the primary component of the fibrillar plaques that characterize AD [11]. It is produced by the sequential cleavage of the amyloid precursor protein (APP) by β- and γ-secretases, via what is known as either the amyloidogenic or β-amyloid pathway [12-14]. Often referred to as an overflow pathway, the amyloidogenic pathway, first cleaves APP extracellularly via a β-secretase producing a C99 peptide, which is subsequently cleaved by the γ-secretase complex resulting in Aβ oligomer production. However, the major pathway by which APP is cleaved is the non-amyloidogenic or α-secretase pathway, in which APP is cleaved by both α- and γ-secretases producing sAPPα and an APP intracellular domain. The products of the non-amyloidogenic pathway are considered to be beneficial and are associated with increased synaptic plasticity and neuronal survival [15]. Interestingly, β-amyloid is present in the brains of non-demented individuals, under normal physiological conditions, and is believed to act as part of a negative feedback loop in the regulation of synaptic plasticity and neuronal survival [14]. However, it is the over production of β-amyloid in AD that likely results in neurodegeneration and cognitive decline [11,16-19].

In addition to its presence in the insoluble fibrillar plaques, which characterize AD, β-amyloid exists in the brain in the form of soluble Aβ oligomers [17]. While early AD research focused on Aβ plaques as the neurotoxic species [20], recent evidence suggests that plaques may actually be a physiological ‘end point’ of limited harmfulness, while soluble oligomeric Aβ isoforms, in particular the Aβ42 fragment, are the primary source of neurotoxicity [21,22]. The accumulation of Aβ oligomers predominantly occurs in brain regions associated with learning and memory, including the hippocampus, and binds to sites that are located at neuronal synapses to cause the disruption to neuronal signaling and ultimately neuronal cell death [23,24]. A number of Aβ oligomer binding sites have been proposed, encompassing, but not limited to, glutamate receptors (both ionotropic and metabotropic), insulin receptors, acetylcholine receptors (both muscarinic and nicotinic), as well as cellular prion protein (PrP^c^) which may function as a co-receptor for Aβ [9,25-28]. Although the precise binding site remains controversial, Aβ oligomers presumably act via multiple receptors at synapse, thereby contributing to the range of issues that characterize AD.

PrP^c^ is a glycosylphosphatidylinositol (GPI)-anchored membrane protein, whose normal cellular function remains unclear [29]. However, PrP^c^ can undergo a conformationally inappropriate folding resulting in scrapie prion protein (PrP^Sc^) that is linked to transmittable spongiform encephalopathies that cause terminal neurodegenerative disorders in both human and animals [30]. Several ligands bind to PrP^c^ including the laminin γ1-chain, Cu^2+^ ions and Aβ42 oligomers [28-30]. Aβ42 oligomers bind to PrP^c^ with nM affinity [25]. PrP^c^ is suggested to be required for the suppression of long-term potentiation (LTP) in hippocampal slices, but LTP has been reported in hippocampal slices derived from PrP^c^ null mice [25].

## Glutamate

Glutamate is the major excitatory neurotransmitter in the brain. Under normal physiological conditions glutamate mediates learning and memory, as well as other higher order integrative brain function, but pathological glutamate signaling is also known to contribute to neuronal cell death [31-33]. Glutamate is released into the synapse following the depolarization of pre-synaptic neurons and is cleared from the synapse by the GLT transporter into astrocytes. Under normal physiological conditions, the removal of glutamate is rapid and neuroprotective [34]. Glutamate exerts its effects via both ionotropic glutamate receptors (iGluR) and metabotropic glutamate receptors (mGluRs). Ionotropic GluRs are ligand-gated ion channels that include the NMDA, α-amino-3-hydroxy-5-methyl-4-isoxazolepropionic acid (AMPA) and kainite receptors that function to mediate the rapid synaptic responses to glutamate [33]. In contrast, mGluRs encompass 8 subtypes and the activation of these receptors by glutamate results in slower, longer-lasting, modulatory alterations in synaptic activity [35].

The dysregulation of and/or pathological signaling of glutamate receptors is well established for a number of neurodegenerative diseases, including AD [35]. Glutamate becomes excitotoxic as the result of overproduction and impaired clearance from synapses [36]. The excess of glutamate appears to chronically activate both ionotropic and metabotropic glutamate receptors resulting in the elevation of intracellular Ca^2+^ associated with neurodegenerative disease that promotes neuronal injury and cell death [37]. In the present review, we will overview the hypothesis that mGluR5 and NMDAR function as extracellular molecular scaffolding proteins, which facilitate the cellular signaling of PrP^c^/Aβ oligomer complexes in the pathogenesis of AD. These PrP^c^/Aβ oligomer interactions with glutamate receptors represent a novel target for the prevention and/or attenuation of AD progression.

## Metabotropic glutamate receptors

Metabotropic glutamate receptors are members of the G protein coupled receptor superfamily. There are eight mGluR subtypes, which are further divided into 3 groups based on sequence homology, G protein-coupling specificity and pharmacological profile [38]. The Group I mGluRs, mGluR1a and mGluR5a activate phospholipase C to generate both inositol 1,4,5 trisphosphate and diacylglycerol resulting in the release of intracellular Ca^2+^ stores and the activation of protein kinase C [38]. Group I mGluRs, in particular mGluR5, have been specifically implicated in neurodegenerative diseases such as AD, Parkinson’s disease and Huntington’s disease [35,39,40]. Interestingly, Group I mGluRs and their interacting proteins have the ability to function in both a neuroprotective and neurotoxic manner. For example, in a rat model of ischemia, mGluR5 antagonists and agonists, were found to have neuroprotective effects [41]. Group I mGluRs associate with and/or activate Homer, ERK1/2, AKT and mTOR signaling pathways [42-46]. These pathways are considered to be neuroprotective as they are important for cell survival, growth and proliferation, in addition to synaptic plasticity. For example, mGluR1a/Homer/PIKE protein interactions have been demonstrated to contribute to the activation of PI3K and functions to prevent neuronal apoptosis [46]. Furthermore, Group I mGluRs have been shown to modulate the activity of ligand-gated ion channels including the NMDAR and play an important role in regulating both long-term potentiation (LTP) and long-term depression (LTD), which represent cellular mechanisms involved in both the positive and negative regulation of synaptic strength, respectively [47,48]. Similarly, NMDAR activation increases mGluR5 activity [49,50]. Thus, mGluR5 activity is also associated with excitotoxic cell death, caused by elevated intracellular Ca^2+^ by virtue of the ability of mGluR5 to potentiate NMDAR function [48]. mGluR5 has also been linked to the RNA binding protein fragile X mental retardation protein (FMRP), which is known to repress protein synthesis at the synapse [51,52]. APP is among the proteins regulated by FMRP [51]. The activation of mGluR5 has been shown to increase FMRP mediated translation of APP, which could lead to neuroprotection under normal physiological conditions or excessive Aβ production in animal models of AD [53].

In the human AD brain, mGluR1 expression has been found to be reduced, with the reduced level of mGluR expression being correlated to the severity of the disease, whereas mGluR5 protein expression was unchanged [54]. Interestingly, there is some discrepancy over changes to mGluR5 expression, with some research suggesting that mGluR5 mRNA expression is up-regulated [55] and mGluR5 cell surface expression is increased in a APPswePS1ΔE9 mouse model of AD [10]. Additionally, elevated expression of mGluR5 has been observed in astrocytes, most notably with this elevation is observed in astrocytes found clustered around Aβ plaques [54]. However, mGluR5 represents a candidate receptor contributing to the underlying pathogenesis associated with AD [9,10,27]. Differences in mGluR1 and mGluR5 expression, in the AD brain, present the possibility that these receptors play functionally distinct roles in the progression of AD pathology. This has suggested that mGluR1 may play a protective role in AD, whereas altered mGluR5 signaling in AD may be neurotoxic [55]. However, this hypothesis remains to be clearly determined.

## Role of group I mGluRs as scaffolds for the binding of Aβ oligomers and PrP^c^

**Figure 1:**
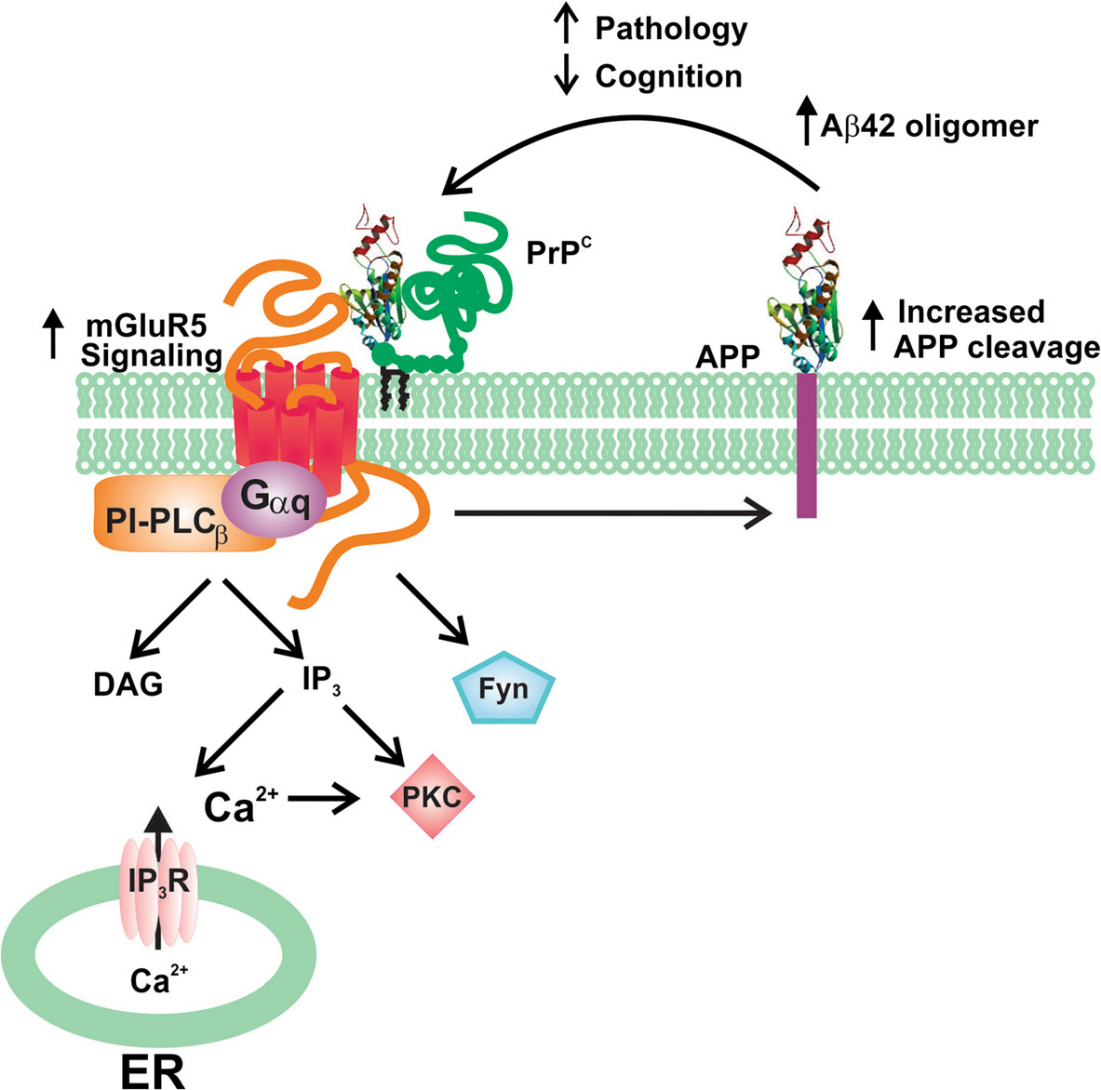
**mGluR5 function as scaffolds for the formation of A** **β** **oligomer and PrP** ^**C**^ **signaling complexes.** The extracellular domain of mGluR5 interacts with both PrP^c^ and Aβ42 which results in the activation of Ca^2+^ release from intracellular stores, PKC translocation and ERK1/2 phosphorylation. Aβ42 and PrP^c^ also activate mGluR5 to stimulate Fyn kinase-mediated and mGluR5 activation has been linked to the regulation of APP protein translation. APP, amyloid precursor protein; DAG, diacylglycerol; ER, endoplasmic reticulum; ERK, extracellular regulated kinase; PLCβ, phospholipase Cβ; PrP^c^, cellular prion protein.

Numerous studies have implicated Group I mGluR signaling in the regulation of Aβ42 toxicity in neurons and in AD mouse models [9,10,27] and Aβ42 oligomers and PrP^C^ increase mGluR5a-dependent LTD *in viv*o [56]. Aβ42 oligomers also induce changes in the subcellular localization of CaMKII resulting in a reduction of AMPARs in the synaptic membrane [57]. Aβ42 peptide is also reported to uncouple mGluR5-dependent activation of PKC, but not ERK1/2 activity [58]. Oligomeric forms of Aβ42 also bind to PrP^c^ to alter synaptic plasticity and recent work clearly supports the concept that PrP^c^ is required for Aβ42 oligomer-mediated neuronal toxicity [25,59-65]. PrP^c^ has been shown to stimulate Ca^2+^ release from neuronal intracellular stores in response to laminin γ1-chain peptide-dependent activation of endogenous PrP^c^, as well as stimulate PKC translocation to the plasma membrane in response to either mGluR1a or mGluR5a activation [28]. Laminin γ1-chain peptide-mediated Ca^2+^ release from neurons is antagonized by either mGluR1a or mGluR5a antagonists and is not observed in primary neurons derived from PrP^c^ null mice [28]. More recently, mGluR5, but not mGluR1, has been suggested to be the primary co-receptor for both PrP^c^ and Aβ oligomers [9] (Figure 1). Specifically, convincing data from the Stritmatter laboratory indicates that mGluR5, but not an extensively investigated battery of other GPCRs (including mGluR1), functions as the extracellular scaffolding protein receptor that is essential for the regulation of Aβ oligomer and PrP^c^ signaling in AD [9]. However, a potential role for mGluR1 cannot be dismissed given its role in laminin γ1-chain peptide-mediated Ca^2+^ release [28]. The report that mGluR5 functions as a receptor for both Aβ oligomers and PrP^c^ is consistent with a previous study showing that Aβ oligomers stimulate the lateral diffusion and clustering of mGluR5 at synapses [27]. This ultimately results in increased Ca^2+^ release from intracellular stores ultimately resulting in synaptic deterioration [27]. Whether this Aβ oligomer-stimulated clustering contributes to mGluR5- and/or mGluR1-dependent alterations in LTP and LTD remains to be determined. Nevertheless, the seminal study by Stritmatter and colleagues provides evidence that mGluR5 plays an integral role in regulated Aβ oligomer pathology, as the deletion of mGluR5 expression reduced Aβ oligomer-mediated synapse loss and mGluR5 antagonist (MTEP) treatment results in the amelioration of cognitive deficits normally observed APPswePS1ΔE9 mice at 9 months of age [9]. Consistent with these observations, we have recently shown that the genetic deletion of mGluR5 in APPswePS1ΔE9 mice also results in the reversal of the cognitive deficits associated with this mouse model of AD at both 9 and 12 months of age, and that soluble Aβ oligomer levels and Aβ plaques in APPswePS1ΔE9 mice are reduced in the absence of mGluR5 expression at 12 month of age [12]. More recently, it has been shown that mGluR5 allosteric modulators can disrupt Aβ oligomer-stimulated interactions between mGluR5 and PrP^C^ suggesting that it may be possible to develop new compounds to interrupt the pathogenesis observed in AD [66]. The observation that mGluR5 activation increases FMRP-mediated translation of APP, suggests the possibility that Aβ oligomer stimulation of mGluR5 may leaded to increased Aβ production resulting in a positive feedback loop the exacerbates AD pathology [53] (Figure 1).

Dysregulation of Group I mGluRs may also further exacerbate AD progression, as a consequence of bidirectional cross talk with NMDARs, as NMDAR activation may increase mGluR5 signaling [49,50]. This cross activity between mGluR5 and NMDARs involves reduced mGluR5 desensitization in response to GRK2 and is also due to NMDAR-stimulated dephosphorylation of mGluR5 and Homer protein regulated cross-regulation of AMPAR activity [49,55,67]. This likely contributes to Group I mediated regulation of NMDAR-dependent LTP and LTD [40,68].

## NMDARS

In the previous sections, we have discussed mGluRs as scaffolds for PrP^c^ and β-amyloid in AD. However, glutamate also plays a central role in regulating synaptic transmission by activating ionotropic receptors, namely AMPA, kainate and NMDA receptors [69]. There is new evidence that NMDARs are regulated by both PrP^c^ and Aβ oligomers [70]. NMDARs are activated by glutamate, as well as the synthetic agonist NMDA, and the co-agonists glycine and D-serine which function to modulate glutamate-dependent activation of NMDARs resulting in the opening of a cation channel that is permeant to both Ca^2+^ and Na^+^ [71]. This has two major consequences, first, it creates an electrogenic response that induces an excitatory postsynaptic potential, and second, Ca^2+^ entering via NMDARs triggers cytoplasmic signaling cascades that eventually culminate in altered cellular activity, such as changes in gene expression. While NMDARs are important for learning and memory [72,73], their dysfunction is linked to pathophysiological signaling that is associated with neurodegenerative diseases such as AD [74-76]. Glutamate binding to NMDARs mediates NMDAR channel opening, whereas the dissociation of the ligand causes channel closure (or deactivation) [77]. NMDARs also undergo a process called desensitization in the prolonged presence of receptor agonist [78], which is designed to protect cells from Ca^2+^ overload. This desensitization process is slowed in a dose-dependent manner by the co-agonist glycine [78,79]. NMDARs are potently regulated by extracellular Mg^2+^ ions, which occlude the channel pore at hyperpolarized potentials, and this block is relieved following membrane depolarization of the post-synaptic neuron [80,81]. Other metal ions that regulate NMDARs include Zn^2+^ and Cu^2+^ [82-85].

## PrP^c^ interactions with NMDARs

NMDARs are functionally modulated by both PrP^c^ and PrP^Sc^, both *in vivo* and *in vitro* [70]. For example, PrP^c^ null mice exhibit depressive-like behaviours [86] and reduced pain thresholds [87], which are reversed by delivery of the NMDA receptor antagonist MK-801. Moreover, cell death of neuronal cultures infected with PrP^Sc^ is partially abrogated by MK801 treatment [88]. NMDAR activity is also enhanced in PrP^c^ null neurons [89]. Field potential recordings from hippocampal slices exhibit an increased number of population spikes that are exacerbated by the removal of extracellular Mg^2+^, and are prevented by the NMDAR blocker APV. In PrP^c^ null neurons there is also an increase in the amplitude and duration of the miniature synaptic NMDA currents. Also, in PrP^c^ null neurons there is a drastic slowing of the deactivation kinetics of NMDAR currents and direct delivery of NMDA into the brains of PrP^c^ null mice increases lesion size compared to WT animals [89]. This suggests a mechanism by which PrP^c^ regulates subunit stoichiometry between GluN1 and various types of GluN2 subunits to account for divergent effects on NMDAR function [70]. In this regard, it is noteworthy that NMDARs can be co-immunoprecipitated with PrP^c^ from brain homogenate [85,89], suggesting that PrP^c^ forms a molecular complex with NMDARs and may function as a NMDAR ligand, rather than regulating NMDAR function solely via second-messenger signal transduction pathways.

More recently, we have described a type of PrP^c^-mediated modulation of NMDAR activity that is dependent upon Cu^2+^ ions [85]. PrP^c^ is a high-affinity Cu^2+^ binding protein that contains five Cu^2+^ binding sites that exhibit varying Cu^2+^ binding-affinity [90-92]. When Cu^2+^ ions are chelated by the exogenous application of either cuprizone or bathocuproinesulfonate (BCS), NMDAR current amplitudes are increased and receptor desensitization is slowed, leading to a persistent current [85]. A similar slowing of NMDAR desensitization is also observed in neurons either derived from PrP^c^ null mice or upon acute enzymatic cleavage of PrP^c^. Importantly, BCS has no further effect on NMDAR desensitization in PrP^c^ null neurons. Compared with wild type neurons, the glycine concentration-dependence of the magnitude of the persistent NMDAR currents is shifted leftward in PrP^c^ null neurons. Hence, over a wide range of glycine concentrations, there is an increase in the non-desensitizing current component. Based on these findings, we conclude that in the presence of PrP^c^, glycine affinity for the NMDAR is reduced and thus the receptor desensitizes more rapidly. Thus, BCS-mediated chelation of copper appears to weaken the biochemical interactions between PrP^c^ and the NMDAR complex [85] suggesting that this PrP^c^/NMDAR complex is copper-ligand regulated.

## Aβ-mediated regulation of NMDA receptor function

Numerous studies have linked NMDARs to AD and the clinically utilized AD drug, memantine, is a known allosteric blocker of NMDARs [93-96]. In cultured neurons Aβ42 oligomers appear to initiate spontaneous NMDAR currents and NMDAR internalization [97,98] and a number of studies have shown that the molecular mechanisms underlying Aβ42-dependent alterations in NMDAR activity and trafficking are complex [97,99]. It has been reported that Cu^2+^ binds to Aβ42 with an attomolar affinity-constant [100]. In line with what we have described above, the application of either nM concentrations of Aβ42 oligomers, or application of μM concentrations of Aβ42 monomers, mimics the effect of BCS on NMDAR function [70], yielding NMDA currents that exhibit reduced desensitization. Based on our findings, we propose a mechanism by which binding of Aβ42 oligomers at nM concentrations bind directly to PrP^c^ and alter PrP^c^-mediated NMDAR desensitization, whereas Aβ42 monomers, when present in the low μM range, might in manner analogous to BCS, simply chelate Cu^2+^, thus leading to PrP^c^ dissociation from the NMDAR [70]. This mechanism may also help to account for the disparate findings by various groups who have investigated the role of Aβ oligomer/PrP^c^ complexes, in regulating reduction in LTP mediated by both mGluRs and NMDARs in wild-type mice and AD mouse models [25,40,59,60,65].

It is important to note that one of the consequences of slowed NMDAR desensitization is the potential for persistent calcium entry following prolonged glutamate activation resulting in neuronal cell death [70]. However, if the elimination of PrP^c^ expression results in slowed NMDAR desensitization kinetics that are analogous to that seen in the presence of Aβ42 oligomers, then one would also expect to observe AD-like pathology in PrP^c^ null mice, but this is not the case. They key to answering this conundrum likely lies in the fact that a slowing of desensitization kinetics manifests itself pathologically only when there is prolonged excess of glutamate agonist. In PrP^c^ null mice, synaptic glutamate is likely cleared quickly by reuptake GLT-1 transport mechanisms on astrocytes, and thus NMDARs are only briefly activated at synapses. In contrast, in mouse models of AD, it is likely that glutamate reuptake mechanisms are perturbed by Aβ42 oligomers, leading to the buildup of synaptic and extra-synaptic glutamate that triggers prolonged activation of both NMDAR- and mGluR-mediated calcium entry resulting in cell damage that may contribute to synaptic loss in AD [101]. In this context it is important to note that extrasynaptic GluN2B containing NMDA receptors are thought to be the primary drivers of excitotoxicity, whereas synaptic GluN2A receptors were found to be neuroprotective [102-104]. On the other hand a recent study has linked the degenerative effects of Aβ to a dysregulation of GluN2A containing receptors [105]. Precisely how the interactions with the NMDAR complex occur in a NMDAR subtype specific manner remains to be determined. Taken together, there is increasing body of experimental evidence suggesting that PrP^c^ is an important regulator of NMDAR functions that are regulated by Cu^2+^ ions. Aβ peptides (in both their monomeric and oligomeric forms) appear to mediate dysregulation of NMDAR-mediated synaptic function, thereby potentially contributing to AD pathology. These processes may also be further complicated by hyperphosphorylated tau protein and Fyn kinase, which are also closely linked to NMDARs and PrP^c^ activity, but beyond the scope of the present review [105-109].

## Summary

In summary, mGluR5 and NMDARs represent potential therapeutic targets for the treatment of AD. Particularly encouraging, are recent studies in which mouse models of AD were treated with the mGluR5 antagonist MTEP, and memory was rescued in the Morris water maze (MWM) a spatial memory behavioural paradigm [10]. Additionally, in an AD mouse model, in which mGluR5 was genetically deleted, mice showed improved memory in the MWM, as well as a substantial reduction in Aβ plaques and oligomers [12]. Of the drugs available for the treatment of AD, memantine, is a known allosteric blocker of NMDARs [93-96]. Although memantine is of limited therapeutic value, the modulation of NMDAR function may nonetheless offer the possibility for effective target for the treatment of AD. It is likely that the effects of Aβ42 oligomers and PrP^c^ on mGluR5 and NMDAR signaling do not occur in isolation as Aβ42 oligomer binding to PrP^c^ and mGluR5 results in the activation of Fyn kinase to regulate signaling downstream of mGluR5 [10] (Figure 1). This activation of Fyn results in the phosphorylation of the GluN2B subunit of the NMDAR that ultimately results in a loss of cell surface NMDARs [10]. Of particular interest will be to determine whether the antagonism of Aβ42 oligomer and PrP^c^ interactions with both mGluR5 and NMDARs will serve as an effective treatment for AD.

## Abbreviations

AD: Alzheimer’s disease
NFT: Neurofibrillary tangles
Aβ: Beta amyloid
ROS: Reactive oxygen species
APP: Amyloid precursor protein
PrP^c^: Cellular prion protein
LTP: Long term potentiation
LTD: Long term depression
mGluR: Metabotropic glutamate receptor
iGluR: Ionotropic glutamate receptor
NMDA: N-methyl-D-aspartate
AMPA: α-amino-3-hydroxy-5-methyl-4-isoxazolepropionic acid
GPCR: g protein coupled receptor
PLC: Phospholipase C
DAG: Diacylglycerol
mTOR: Mammalian target of rapamycin
CNS: Central nervous system
PS1: Presenilin 1
FMRP: Fragile X mental retardation protein
